# Fucoidan treatment alleviates chilling injury in cucumber by regulating ROS homeostasis and energy metabolism

**DOI:** 10.3389/fpls.2022.1107687

**Published:** 2022-12-23

**Authors:** Duo Lin, Ruyu Yan, Mengying Xing, Shuyuan Liao, Jinyin Chen, Zengyu Gan

**Affiliations:** Jiangxi Key Laboratory for Postharvest Technology and Nondestructive Testing of Fruits and Vegetables, Collaborative Innovation Center of Postharvest Key Technology and Quality Safety of Fruits and Vegetables, Jiangxi Agricultural University, Nanchang, China

**Keywords:** cucumber fruit, chilling injury, fucoidan, antioxidant capacity, energy metabolism

## Abstract

**Introduction:**

Chilling injury is a major hindrance to cucumber fruit quality during cold storage.

**Methods and Results:**

In this study, we evaluated the effects of fucoidan on fruit quality, reactive oxygen species homeostasis, and energy metabolism in cucumbers during cold storage. The results showed that, compared with the control cucumber fruit, fucoidan-treated cucumber fruit exhibited a lower chilling injury index and less weight loss, as well as reduced electrolyte leakage and malondialdehyde content. The most pronounced effects were observed following treatment with fucoidan at 15 g/L, which resulted in increased 1,1-diphenyl-2-picrylhydrazyl and hydroxyl radical scavenging rates and reduced superoxide anion production rate and hydrogen peroxide content. The expression and activity levels of peroxidase, catalase, and superoxide dismutase were enhanced by fucoidan treatment. Further, fucoidan treatment maintained high levels of ascorbic acid and glutathione, and high ratios of ascorbic acid/dehydroascorbate and glutathione/oxidized glutathione. Moreover, fucoidan treatment increased the activities of ascorbate peroxidase, monodehydroascorbate reductase, dehydroascorbate reductase, and glutathione reductase and their gene expression. Fucoidan treatment significantly delayed the decrease in ATP and ADP, while preventing an increase in AMP content. Finally, fucoidan treatment delayed the decrease of energy charge and the activities and gene expression of H^+^-ATPase, Ca^2+^-ATPase, cytochrome c oxidase, and succinate dehydrogenase in cucumber fruits.

**Conclusion:**

Altogether, our findings indicate that fucoidan can effectively enhance antioxidant capacity and maintain energy metabolism, thereby improving cucumber cold resistance during cold storage.

## 1 Introduction

Cold storage is a common method of preserving horticultural products after harvest. However, for cold-sensitive horticultural products, such as cucumber (Wang et al., 2018; Wang et al., 2020a), peach (Song et al., 2022), and kiwifruit (Liu et al., 2023), low temperatures can cause chilling injury (CI). CI damages the permeability of the fruit cell membrane, leading to cytoplasmic leakage. When cucumber fruit is transferred from cold storage to room temperature, the symptoms of CI appear quickly, manifested as watery spots on the fruit epidermis and an unpleasant odor, which are generally deemed unacceptable by consumers (Tsuchida et al., 2010; Wang et al., 2018).

Cold stress induces the accumulation of reactive oxygen species (ROS) in plant tissues, leading to a series of oxidative damage events, such as membrane lipid peroxidation, and eventually inducing cell death (You and Chan, 2015; Mittler et al., 2022). The plant ROS-scavenging system consists of an enzymatic defense system and a non-enzymatic antioxidant defense system (Gill and Tuteja, 2010). The former includes peroxidase (POD), catalase (CAT) and superoxide dismutase (SOD). SOD can remove excess superoxide anions ( ${\text{O}}_{2}^{-}$ ) in plants and reduce the damage caused by ${\text{O}}_{2}^{-}$ to the cell membrane. In turn, POD and CAT catalyze the decomposition of hydrogen peroxide (H_2_O_2_) to maintain H_2_O_2_ at a low level, minimize the production of hydroxyl radicals (-OH), and protect cell membrane structure (Yu et al., 2022). The ascorbic acid-glutathione (AsA-GSH) cycle is an important component of the non-enzymatic antioxidant system. The AsA-GSH cycle comprises ascorbate peroxidase (APX), monodehydroascorbate reductase (MDHAR), dehydroascorbate reductase (DHAR), and glutathione reductase (GR), which can directly remove H_2_O_2_ from plant tissues. The AsA-GSH cycle promotes the production of two non-enzymatic antioxidants (AsA and GSH) to maintain the balance of redox substances in plants (Peng et al., 2022). The ROS-scavenging system has been shown to be critical for the enhancement of cold resistance in many horticultural products, such as loquats (Cai et al., 2011), bell peppers (Endo et al., 2019), okra (Li et al., 2023), and bananas (Chen et al., 2021b). Therefore, improving antioxidant capacity is a promising approach for alleviating low temperature-induced oxidative damage in horticultural products.

Mitochondria are important sites for respiration and the production of ATP in living tissues, thus providing energy for all cellular activities (Chen et al., 2021a). H^+^-ATPase, Ca^2+^-ATPase, cytochrome c oxidase (CCO), and succinate dehydrogenase (SDH) are key enzymes involved in energy synthesis in the mitochondrial membrane (Li et al., 2021a). The decrease or loss of activity of these enzymes can lead to insufficient energy supply to cells, which in turn leads to cellular senescence and death. Numerous studies have shown that the occurrence of CI during the post-harvest cold storage of horticultural products, such as strawberries (Li et al., 2018), pears (Tao et al., 2019), and peaches (Jin et al., 2013), is related to energy metabolism. Energy loss leads to the accumulation of ROS, cell membrane damage, impaired metabolism, and a slow response to low temperature signals, thus accelerating the occurrence of CI.

Numerous preservation techniques, such as brief exposure to hot water (Nasef, 2018), pre-storage cold acclimation (Wang et al., 2018; Wang et al., 2021a), or treatment with hydrogen sulfide (Wang et al., 2022), melatonin (Madebo et al., 2021), or glycine betaine (Zhang et al., 2008), have been shown to extend the shelf life of cold-stored cucumber fruits. Polysaccharide coatings have also been used to improve cold resistance after harvest; for example, chitosan has been shown to improve cold resistance in cucumber fruit (Ru et al., 2020), and treatment with *Astragalus* polysaccharides inhibited the occurrence of CI in bananas (Tian et al., 2022). Fucoidan is a type of fucose-enriched and sulfated polysaccharide that extracted from different kinds of brown seaweed, which contains L-fucose and sulfate groups (Zhao et al., 2004). As a nontoxic natural heteropolysaccharide, fucoidan has multiple biological functions, such as anti-coagulation, anti-tumor, anti-thrombus, anti-virus, anti-oxidation and enhance the body’s immune function (Senthilkumar et al., 2013; Wu et al., 2016). In addition, fucoidan can be used as a preservative for storing fruit and vegetables after harvest. Some studies have shown that fucoidan improves antioxidant capacity and maintains post-harvest fruit quality; indeed, fucoidan can extend the shelf life of some fruits, such as mangos (Xu and Wu, 2021), and strawberries (Duan et al., 2019; Luo et al., 2020). However, to date, the effect of fucoidan on CI in cucumber fruits has rarely been reported. Therefore, here, we investigated whether fucoidan treatment can improve cold resistance in cucumbers stored at low temperature, with a focus on ROS homeostasis and energy metabolism. Our findings support the use of fucoidan as a new preservative for cucumber cold storage.

## 2 Materials and methods

### 2.1 Plant materials and treatments

Cucumbers (*Cucumis sativus* cv. Sunstar) were collected during the commercial harvest period (approximately 10 d after flowering) from a farm in Nanchang, Jiangxi, China. Fruits were washed with distilled water and dried naturally. Fruits of uniform size and without any disease or defects were randomly separated into four groups and coated with 0 (control), 5, 10, or 15 g/L fucoidan (extract from brown algae, medium molecular weight, purity≥95%, Baichuan, Xi’an, China). Treatments were performed in triplicates comprising 300 fruits for each replicate. Treated fruits were packed in 0.01 mm thick polyethylene plastic bags and stored for 12 days at 4°C and 90% relative humidity. For each replicate, 20 fruits were collected every three days, 15 of which were placed at 20°C for two days to observe CI, and the pulp (without peel tissue and internal seeds) of the remaining 5 fruits was collected at random and stored at -80°C after quick freezing under liquid nitrogen.

### 2.2 Measurement of CI, weight loss, electrolyte leakage, and malondialdehyde content

For CI: The degree of cucumber CI was divided into five levels. 0 = no CI, 1 = 0< CI area ≤ 25%, 2 = 25%< CI area ≤ 50%, 3 = 50%< CI area ≤ 75%, 4 = 75%< CI area ≤ 100%. CI inedx was calculated as follows:


$$\text{CI index}=\frac{\text{Σ }\left(\text{CI level }\times \text{number of fruit at that level}\right)}{4\;\times \text{total fruit}}$$

Ten tissue discs with uniform size (thickness: 1 mm, diameter: 8 mm) from five cucumber fruits were prepared for the measurement of electrolyte leakage. Electrolyte leakage, and malondialdehyde (MDA) content of the experimental cucumber fruits were measured as previously described (Wang et al., 2022).

For weight loss: five cucumber fruits were weighed using an electronic scale at a time, and the measurements were repeated three times. Weight loss was calculated as follows:


$$\text{Weight loss (}\%=\frac{\text{initial fruit weight }-\text{ fruit weight at day }12}{\text{initial fruit weight}}\times \;100\%$$

### 2.3 Scavenging rates of free radicals, ${\text{O}}_{2}^{-}$ production rate, and H_2_O_2_ content

One gram of pulp from control and fucoidan-treated cucumber fruits was used for the measurement of 1,1-diphenyl-2-picrylhydrazyl (DPPH) scavenging rate, -OH scavenging rate, ${\text{O}}_{2}^{-}$ production rate, and H_2_O_2_ content using a DPPH scavenging rate detection kit (BC4750, Solarbio, Beijing, China), -OH scavenging rate detection kit (BC1320, Solarbio, Beijing, China), ${\text{O}}_{2}^{-}$ content detection kit (BC1290, Solarbio, Beijing, China), and H_2_O_2_ content detection kit (BC3595, Solarbio, Beijing, China), respectively. The unit percentage (%) represents the DPPH and -OH scavenging rates. The ${\text{O}}_{2}^{-}$ production rate and H_2_O_2_ content are expressed as μmol s^-1^ kg^-1^ and mmol kg^-1^, respectively.

### 2.4 Determination of antioxidant and ascorbic acid-glutathione cycle enzyme activities

To quantify POD, SOD, and CAT activities, 1.0 g of pulp from control and fucoidan-treated cucumber fruits was homogenized with 5 ml of 50 mM pre-cooled sodium phosphate buffer (pH 7.5) containing 5 mM dithiothreitol and 5% polyvinylpyrrolidone. The mixture was then centrifuged at 12,000 g for 15 min at 4°C and the supernatant was collected. POD, SOD, and CAT activities were determined as previously described (Chen et al., 2022), and expressed as U kg^-1^. One U of POD and CAT activities represents an absorbance change of 0.01 per gram of sample per minute at 470 nm and 240 nm, respectively. In turn, 1 U of SOD activity represents the amount of SOD per gram of sample in 1 mL of reaction solution when SOD inhibition reached 50%.

The activities of APX, MDHAR, DHAR, and GR were determined using the appropriate test kits (BC0220 for APX activity, BC0650 for MDHAR activity, BC0660 for DHAR activity, and BC1160 for GR activity; Solarbio, Beijing, China) with absorbance measured at 290, 340, 412, and 340 nm, respectively. Enzyme activities are expressed as U kg^-1^, where 1 U represents 1 μmol of enzyme oxidized per gram of sample per minute.

### 2.5 Determination of AsA, DHA, GSH, and GSSG content

One gram of homogenized cucumber fruit was mixed with 15 mL of pre-cooled 5% (w/v) trichloroacetic acid and centrifuged at 12,000 rpm for 20 min at 4°C. The supernatant was collected and AsA and DHA content (mg kg^-1^) was determined as previously described (Zhang et al., 2021). DHA content was calculated as follows:


DHA content=total AsA content – AsA content

GSH and GSSG contents (mg kg^-1^) were detected using the appropriate test kits (BC1170 for GSH content; BC1180 for GSSG content; Solarbio, Beijing, China) with absorbance measured at 412 nm.

### 2.6 Determination of ATP, ADP, and AMP content and energy charge

One gram of cucumber pulp tissue was ground under liquid nitrogen. Then, 6 mL pre-cooled 0.6 mol·L^-1^ perchloric acid solution was added and the sample was fully homogenized. The homogenate was placed on ice for 10 min and then centrifuged at 8000 rpm at 4°C for 15 min. After collecting the supernatant, the pH was adjusted to 6.8 with 1.0 mol·L^-1^ KOH. The neutralized sample was placed on ice for 30 min and then centrifuged at 8000 g at 4°C for 15 min. The supernatant was filtered through a 0.45 μm microporous membrane for use. ATP, ADP, and AMP contents (mg kg^-1^) were determined by high-performance liquid chromatography (LC-2030 Plus, Shimadzu, Kyoto, Japan) using a C18 column (250 mm × 4.6 mm, 5 μm, Waters) as previously described (Chen et al., 2021a). Energy charge was calculated as follows:


$$\text{Energy charge (\%)}=\;\frac{\text{ATP }+\;0.5\;\times \text{ ADP}}{\text{ATP }+\text{ ADP }+\text{ AMP}}\times \;100$$

### 2.7 Determination of H^+^-ATPase, Ca^2+^-ATPase, CCO, and SDH activities

One gram of pulp from control and fucoidan-treated cucumber fruits was used for measurement of H^+^-ATPase and Ca^2+^-ATPase activities using an ATPase activity test kit (A016-1-2, Jiancheng Bioengineering Institute, Nanjing, China) with absorbance measured at 660 nm. One gram of pulp from control and fucoidan-treated cucumber fruits was used for measurement of CCO and SDH activities as previously described (Chen et al., 2022). All results were expressed as U kg^-1^.

### 2.8 Gene expression assays

0.5 g of pulp from control and fucoidan-treated cucumber fruits was used for RNA extraction and gene expression analysis as previously described (Gan et al., 2021). The 2X M5 HiPer Realtime PCR Super mix kit (Mei5bio, Beijing, China) was used to perform quantitative real-time polymerase chain reaction (qRT-PCR) using gene-specific primers (**Supplementary Table S1**). Relative gene expression levels were calculated using the 2^-ΔΔ^Ct method.

### 2.9 Statistical analysis

Each experiment was repeated in triplicate. Excel software was used to sort the data, and the results are expressed as the mean ± standard deviation (SD). Pairwise comparisons were made using the single factor method in SPSS26.0, and significant differences were analyzed using the least significant difference test and Duncan’s method (P< 0.05 or P< 0.01). Graphs were drawn using GraphPad Prism 8.0.

## 3 Results

### 3.1 Fucoidan treatment alleviates chilling injury in cucumber fruits

**Figure 1:**
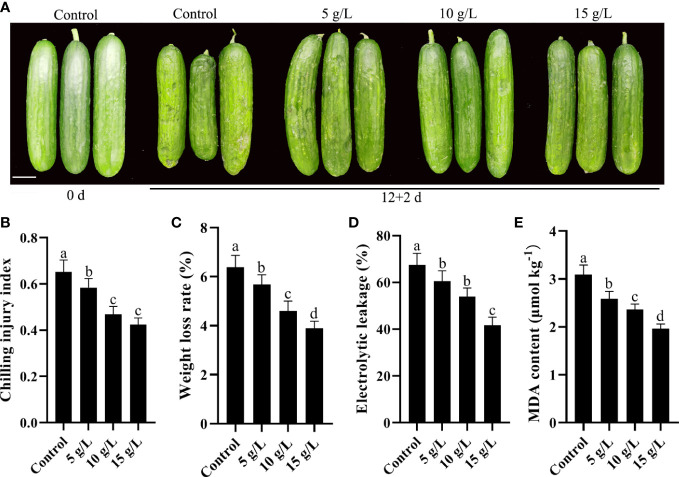
**(A)** Photograph comparing cucumbers treated with 0 (control), 5, 10, or 15 g L^-1^ fucoidan after 12 d of storage at 4°C. **(B-E)** Chilling injury index **(B)**, weight loss rate **(C)**, electrolytic leakage **(D)**, and MDA content **(E)** in cucumber fruit treated with 0, 5, 10, and 15 g L^-1^ fucoidan after 12 d of storage at 4°C. Values are presented as means ± SD of at least three replicates. Bars with different letters are significantly different at P< 0.05, as determined using Duncan’s multiple range tests.

Water-soaked depressions of the epidermis are the most obvious symptom of CI in cucumbers. After 12 days of storage at 4°C, the CI index was 0.65. Compared with controls, coating with 5, 10, or 15 g/L fucoidan decreased the CI index of cold-stored cucumbers (**Figures 1A, B**). Furthermore, weight loss, electrolyte leakage, and MDA content were all reduced following fucoidan treatment (**Figures 1C–E**), reflecting the protective effect of fucoidan against CI. Although all fucoidan treatments reduced CI, treatment with 15 g/L fucoidan achieved the best results; thus, 15 g/L fucoidan was used for subsequent experiments.

### 3.2 ROS scavenging capacity after fucoidan treatment

**Figure 2:**
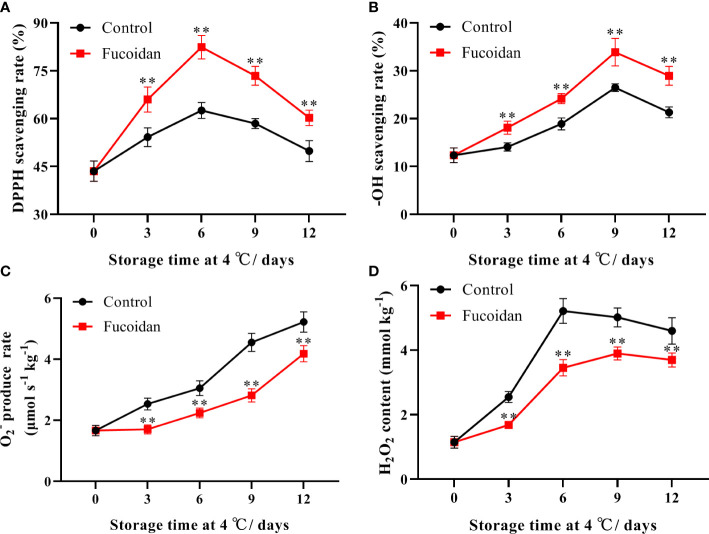
**(A-D)** The DPPH scavenging rate **(A)**, -OH scavenging rate **(B)**, ${\text{O}}_{2}^{-}$ production rate **(C)**, and H_2_O_2_ content **(D)** in fucoidan-treated (15 g L^-1^) and control cucumbers during storage at 4°C. Values are presented as means ± SD of at least three replicates. **P< 0.01.

The DPPH and -OH scavenging rates in cucumbers initially increased and then decreased during cold storage. The DPPH scavenging rate peaked on day 6 of storage, while the -OH scavenging rate peaked on day 9. The DPPH and -OH scavenging rates of fucoidan-treated cucumber fruits were higher than those of control cucumbers (**Figures 2A, B**).

The rate of ${\text{O}}_{2}^{-}$ production increased steadily throughout storage in both control and fucoidan-treated cucumbers. However, the ${\text{O}}_{2}^{-}$ production rate remained lower in the fucoidan-treated group than in the control group throughout the storage period. Control cucumbers exhibited a gradual increase in H_2_O_2_ content, which peaked on day 6 of storage and subsequently declined slightly. In contrast, fucoidan treatment significantly reduced the accumulation of H_2_O_2_ in cold-stored cucumber fruits (**Figures 2C, D**).

### 3.3 Enzyme activity and gene expression of POD, CAT, and SOD after fucoidan treatment

**Figure 3:**
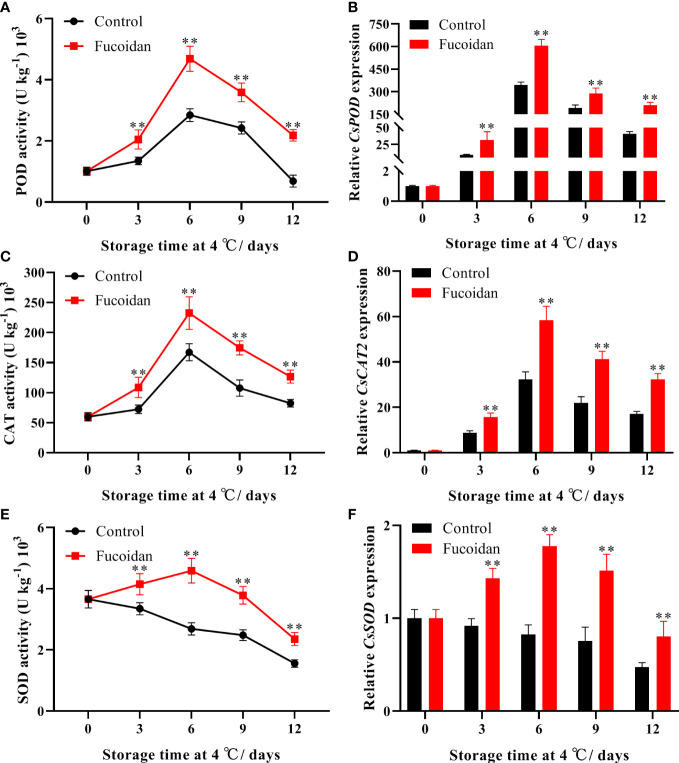
**(A-F)** The enzyme activities and gene expression of POD **(A, B)**, CAT **(C, D)**, and SOD **(E, F)** in fucoidan-treated (15 g L^-1^) and control cucumbers during storage at 4°C. Values are presented as means ± SD of at least three replicates. **P< 0.01.

The activities of the POD and CAT enzymes initially increased in control cucumbers upon cold storage, reaching a maximum on day 6 of storage, and gradually declining thereafter. Conversely, POD and CAT activities increased by 65% and 39%, respectively, following fucoidan treatment, compared to the control group on day 6 of cold storage, and the subsequent decline in these activities was considerably less pronounced than that in the controls (**Figures 3A, C**). In turn, SOD activity in cucumber fruits steadily decreased throughout the storage period, and fucoidan-treated cucumbers maintained higher SOD activity than control cucumbers (**Figures 3E**). Consistent with these results, fucoidan treatment induced the expression of *CsPOD*, *CsCAT2*, and *CsSOD* (**Figures 3B, D, F**).

### 3.4 Changes in AsA-GSH cycle components after fucoidan treatment

**Figure 4:**
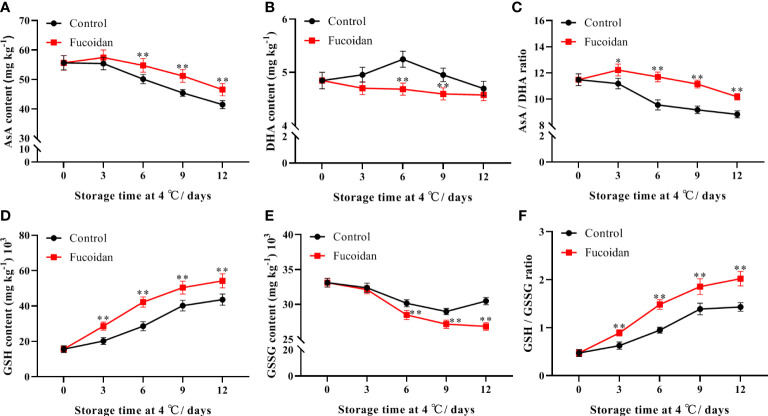
**(A-F)** AsA content **(A)**, DHA content **(B)**, AsA/DHA ratio **(C)**, GSH content **(D)**, GSSG content **(E)**, and GSH/GSSG **(F)** in fucoidan-treated (15 g L^-1^) and control cucumbers during storage at 4°C. Values are presented as means ± SD of at least three replicates. *P< 0.05; **P< 0.01.

The AsA content of cucumber fruits decreased gradually during cold storage in both treated and control fruit groups, but remained higher in the fucoidan-treated group than in the control group on day 6. On day 12, the AsA content of fucoidan-treated fruit was 12% higher than that in control group (**Figure 4A**). In contrast, DHA content in cucumber fruits was not affected by cold storage but was significantly lower in the fucoidan-treated group than in the control group on days 6 and 9 (**Figure 4B**). Although the AsA/DHA ratio decreased gradually during storage, the fucoidan-treated group maintained a higher AsA/DHA ratio than the control group (**Figure 4C**). The GSH content increased with increasing refrigeration time and was higher in fucoidan-treated cucumbers compared to controls throughout the entire storage period (**Figure 4D**). The GSSG content decreased gradually during cold storage, and was significantly higher in the fucoidan-treated group than in the control group from the 6th day of storage (**Figure 4E**). Finally, the GSH/GSSG ratio increased gradually during storage, and the fucoidan-treated group maintained a higher GSH/GSSG ratio than the control group did (**Figure 4F**).

### 3.5 Enzyme activity and gene expression of AsA-GSH cycle components after fucoidan treatment

**Figure 5:**
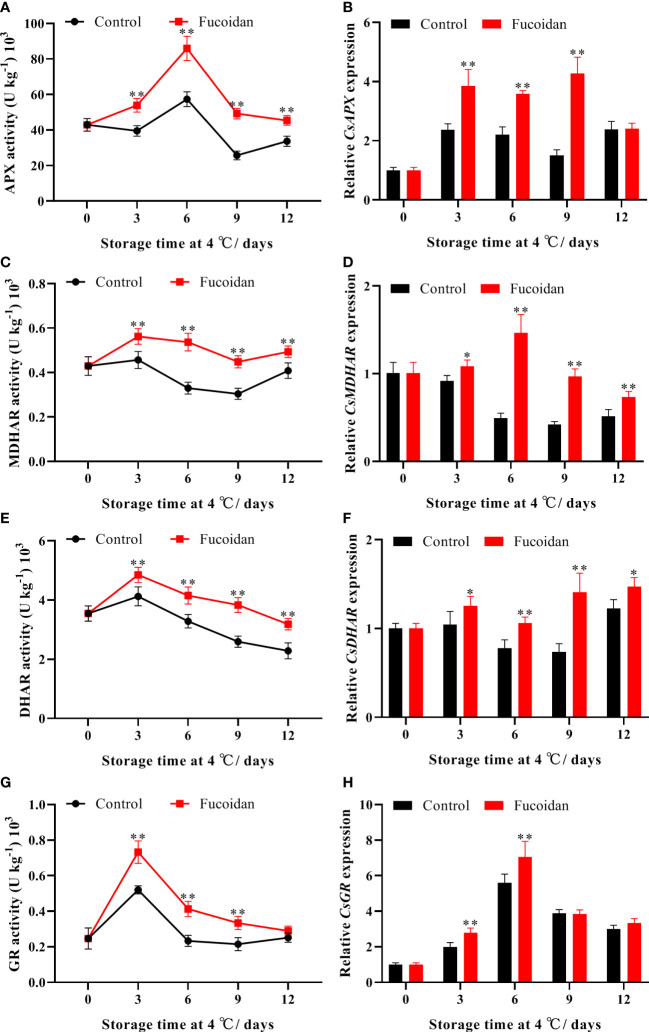
**(A-H)** The enzyme activities and gene expression of APX **(A, B)**, MDHAR **(C, D)**, DHAR **(E, F)**, and GR **(G, H)** in fucoidan-treated (15 g L^-1^) and control cucumbers during storage at 4°C. Values are presented as means ± SD of at least three replicates. *P< 0.05; **P< 0.01.

APX activity initially increased and then decreased during cold storage, reaching a maximum on day 6. The APX activity of fruits in the fucoidan-treated group was significantly higher than that in the control group throughout the storage period (**Figure 5A**). Compared with the control group, MDHAR activity of fruits in the fucoidan-treated group was higher throughout the storage period, becoming 63% higher on day 6 of storage (**Figure 5C**). Similarly, DHAR and GR activities initially increased, reaching a maximum on day 3 before subsequently declining; the activities of both enzymes were significantly improved by fucoidan treatment (**Figures 5E, G**). The expression levels of *CsAPX*, *CsMDHAR*, and *CsDHAR* were consistent with the trends described for enzyme activity, with fucoidan treatment inducing the expression of these genes. Finally, the expression level of *CsGR* in the fucoidan-treated group was significantly higher than that in the control group on days 3 and 6 (**Figures 5B, D, F, H**).

### 3.6 Change in energy levels after fucoidan treatment

**Figure 6:**
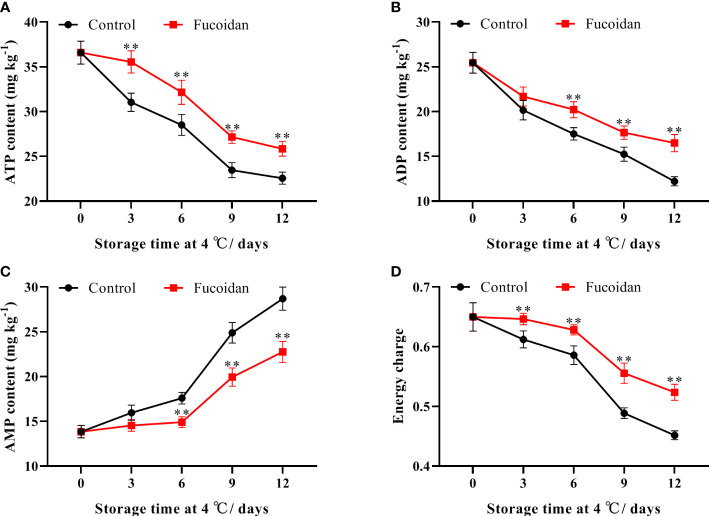
**(A-D)** ATP content **(A)**, ADP content **(B)**, AMP content **(C)**, and energy charge **(D)** in fucoidan-treated (15 g L^-1^) and control cucumbers during storage at 4°C. Values are presented as means ± SD of at least three replicates. **P< 0.01.

The ATP and ADP contents of cucumber fruits declined continuously during cold storage. However, ATP and ADP were significantly higher in fucoidan-treated cucumbers than in control ones throughout the storage period. On day 12, ATP and ADP contents in the treatment group were 14% and 35% higher than those in the control group, respectively (**Figures 6A, B**). Conversely, AMP content in refrigerated cucumber fruits increased gradually during the storage period. Thus, after 6 days of storage, AMP content in fucoidan-treated fruits was significantly lower than that in the control group, decreasing to a level that was 25% lower on day 12 (**Figure 6C**). Consistently, energy charge declined slowly from day 1 to 8 and then declined sharply from the 9th day, with extension of storage time. Compared to control, fucoidan-treated cucumbers maintained a higher energy charge during cold storage (**Figure 6D**).

### 3.7 Enzyme activity and gene expression of energy metabolism-related enzymes after fucoidan treatment

**Figure 7:**
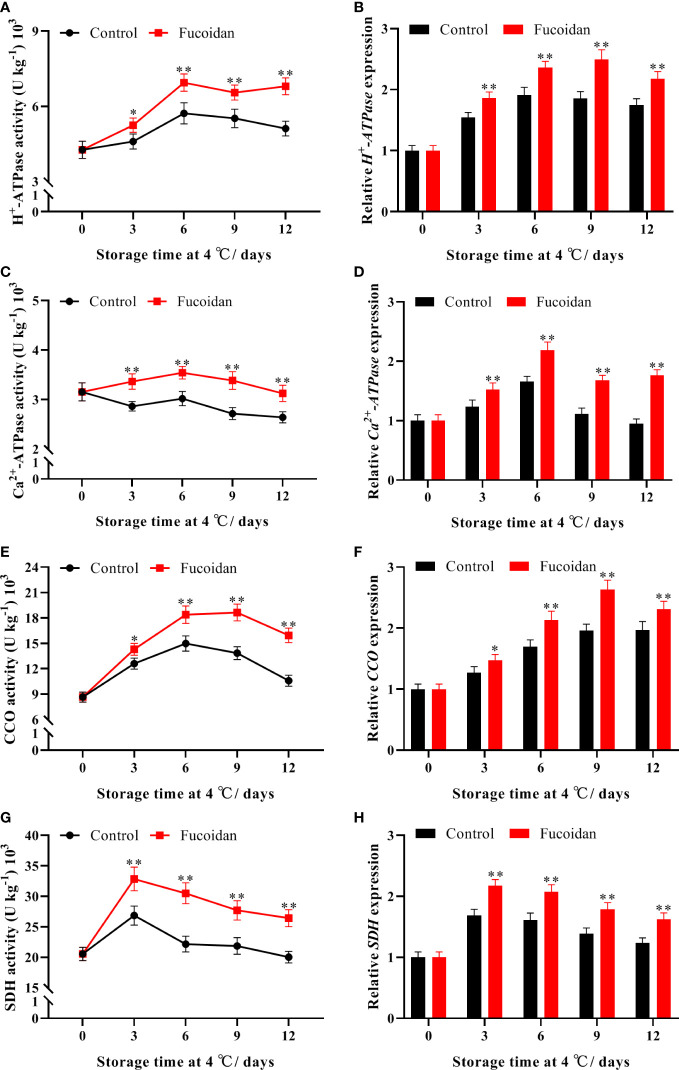
**(A-H)** The enzyme activities and gene expression of H^+^-ATPase **(A, B)**, Ca^2+^-ATPase **(C, D)**, CCO **(E, F)**, and SDH **(G, H)** in fucoidan-treated (15 g L^-1^) and control cucumbers during storage at 4°C. Values are presented as means ± SD of at least three replicates. *P< 0.05; **P< 0.01.

Activity of H^+^-ATPase initially increased and then decreased during cold storage. The activity of H^+^-ATPase in fucoidan-treated cucumbers peaked on day 6 and was 33% higher than that of the control group on day 12 (**Figure 7A**). In contrast, Ca^2+^-ATPase activity remained stable during cold storage, and fucoidan-treated fruit maintained a higher level of activity than control ones (**Figure 7C**). Similarly, CCO activity reached a maximum on day 6 of cold storage before declining. Fucoidan-treated cucumbers maintained a higher level of CCO activity after day 6, and it was 50% higher than that of the control group on day 12 (**Figure 7E**). Additionally, on day 3 of storage, SDH activity of both fucoidan-treated and control fruits peaked, with the former reaching a level 37% higher than that of the control group before beginning to decline (**Figure 7G**). The expression levels of *CsH^+^-ATPase*, *CsCa^2+^-ATPase*, *CsCCO*, and *CsSDH* corresponded with these trends in enzyme activity. Indeed, we found that fucoidan treatment significantly induced the expression of all four genes, thereby regulating the enhancement of their corresponding enzyme activities (**Figures 7B, D, F, H**) and maintaining metabolism in cucumber fruits to preserve normal physiological activities during cold storage.

## 4 Discussion

CI easily occurs in cold-stored cucumber fruits after harvest and leads to a decrease in fruit quality and commodity value (Wang et al., 2018; Madebo et al., 2021; Wang et al., 2022). Polysaccharide coatings are environment friendly materials for preservation and improving cold resistance of horticultural products. Carboxymethyl cellulose (CMC) coating help to reduce CI in pomegranate (Ehteshami et al., 2019) and mandarin fruit (Ali et al., 2021) during cold storage. *Astragalus* polysaccharides delayed the CI and attenuated postharvest peel browning of banana fruit (Tian et al., 2022). In vegetables, the coating of polysaccharide has a similar preservation effect to that of fruit. CMC-based water barrier coating is promising to control the water loss and further maintain the whole quality of pakchoi (Yu et al., 2022). Chitosan induced chilling resistance in cucumber fruit (Ru et al., 2020). Fucoidan has a wide range of biological activities (Zhao et al., 2004)., which has been used to maintain the quality of some fruits. Fucoidan treatment can effectively extend the shelf life of mangoes (Xu and Wu, 2021). Fucoidan treatment improved the cold resistance of strawberry fruit during cold storage (Duan et al., 2019; Luo et al., 2020). In this study, different concentrations of fucoidan (5, 10, and 15 g/L) effectively inhibited CI in cucumber fruits and delayed the appearance of CI symptoms. With the increase of fucoidan concentration, the CI index is lower, the treatment with 15 g/L fucoidan achieved the best results in this study. However, the best result of 15 g/L fucoidan is based on this work, may not the optimum concentration. Thus, the optimum concentration of fucoidan for reducing the CI in cucumber will be further explored.

Cold stress-induced plant damage typically involves an imbalance between ROS production and depletion (Duan et al., 2012); excessive accumulation of ROS, such as H_2_O_2_ and ${\text{O}}_{2}^{-}$ , can irreversibly damage organelles, including chloroplasts and mitochondria. Protecting cells from oxidative damage under stress is considered a major mechanism of plant resistance to low temperatures, and this resistance may depend on the antioxidant system (Mittler et al., 2022). The enzymatic antioxidant system is the main mode of ROS scavenging, and SOD, CAT, and POD are the key antioxidant enzymes involved in this process (Tan et al., 2020). These three antioxidant enzymes work together to reduce the oxidative damage to the cell membrane caused by H_2_O_2_ and O2^-^, thus maintaining its integrity and minimizing CI (Cao et al., 2018; Mirshekari et al., 2020). In this study, fucoidan treatment significantly enhanced the SOD activity of cucumber fruits, effectively reduced the O2^-^ production rate, and maintained high CAT and POD activities, thereby inhibiting the accumulation of H_2_O_2_. Similarly, previous studies have reported that fucoidan treatment enhances antioxidant activity and improves the cold tolerance of strawberries (Duan et al., 2019; Luo et al., 2020). Thus, we conclude that fucoidan treatment is beneficial in regulating the redox state, and improves the antioxidant capacity of cold-stored cucumber fruits.

The AsA-GSH cycle is an important H_2_O_2_ scavenging system in plants. AsA and GSH are important non-enzymatic antioxidants in this cycle, which can scavenge excessive ROS produced by plant cells in response to low-temperature stress injury (Li et al., 2010). The AsA/DHA and GSH/GSSG ratios reflect the redox capacity of plants. Generally, the higher the content of reducing substances, the stronger the stress tolerance of plants (Li et al., 2023). In this study, fucoidan treatment consistently maintained higher AsA and GSH contents and AsA/DHA and GSH/GSSG ratios in cucumber fruits, consistent with results from tomatoes (Li et al., 2019), and bell peppers (Endo et al., 2019). GSH/GSSG ratios play an important role in cold tolerance of tomatoes (Li et al., 2019). Hot water treatment enhanced AsA-GSH cycle to alleviate CI in pepper fruit during postharvest cold storage (Endo et al., 2019). APX, MDHAR, DHAR, and GR are key enzymes involved in AsA-GSH metabolism that cooperate to scavenge excess H_2_O_2_ in cells and convert it into H_2_O and O_2_- (Li et al., 2010). Enhanced gene expression and activity of APX, MDHAR, DHAR, and GR has been shown to improve fruit cold tolerance and reduce the occurrence of CI in bell peppers (Wang et al., 2016; Endo et al., 2019), peaches (Song et al., 2016), okra (Li et al., 2023), and bananas (Chen et al., 2021b). These results support our findings that fucoidan treatment can regulate the activities of key enzymes and the expression of related genes in the AsA-GSH cycle of cucumber fruits during cold storage, thereby improving H_2_O_2_scavenging, reducing the degree of membrane lipid peroxidation, and delaying the occurrence of CI in cucumber fruits.

Energy molecules, such as ATP, play an important role in maintaining the integrity of cell membranes (Saquet et al., 2003). Energy deficits can impair the integrity of cell membranes and lead to a decline in the post-harvest quality of horticultural products (Wang et al., 2020c; Li et al., 2021a). ATP and ADP contents in loquats (Jin et al., 2015), kiwifruit (Wang et al., 2020b), and peaches (Hu et al., 2023), have been reported to drop rapidly and remain at low levels during cold storage. In this study, ATP, ADP, and energy charge declined rapidly in cucumber fruits during cold storage, and the symptoms of fruit CI were aggravated. Fucoidan treatment maintained higher levels of ATP, ADP, and energy charge in cucumber fruits, thereby reducing the ROS-induced membrane damage and alleviating CI. Mitochondria are responsible for energy synthesis; SDH, CCO, H^+^-ATPase, and Ca^2+^-ATPase are key respiratory enzymes located in the inner mitochondrial membrane, and their activities reflect the energy production status of mitochondria (Li et al., 2021b; Wang et al., 2021b). H^+^-ATPase catalyzes ATP synthesis by establishing an H^+^ electrochemical gradient on both sides of the cell membrane to generate transmembrane proton propulsion (Azevedo et al., 2008). Ca^2+^-ATPase is a Ca^2+^ pump responsible for transporting Ca^2+^ into the mitochondria, and a decrease in Ca^2+^-ATPase activity leads to the accumulation of Ca^2+^ in the cytoplasm, leading to the destruction of membrane structural integrity (Chung et al., 2000). SDH catalyzes the conversion of succinic acid to fumaric acid in the tricarboxylic acid cycle, leading to the production of H^+^ and ATP (Ackrell et al., 1978). CCO provides energy to cells mainly through oxidative phosphorylation and is a key enzyme in the oxidative phosphorylation process in the mitochondrial respiratory chain (Millar et al., 1995). Cold stress affects the production of ATP by affecting mitochondrial structure and the activity of these enzymes, and the loss of ATP impairs metabolism and destroys cell structure; this further aggravates CI, resulting in a decline in the quality of horticultural products (Jin et al., 2015; Wang et al., 2020b; Hu et al., 2023). Altogether together, the results of this study demonstrate that fucoidan treatment can maintain mitochondrial function by improving the activities of relevant enzymes in cucumbers during cold storage, thereby ensuring continual energy synthesis and ultimately reducing the occurrence of CI.

## 5 Conclusion

Fucoidan treatment effectively reduced CI in cucumber fruits by modulating ROS scavenging and energy metabolism. Fucoidan treatment can reduce the accumulation of H_2_O_2_ and ${\text{O}}_{2}^{-}$ and the degree of membrane lipid peroxidation by increasing the activity of antioxidant enzymes and the expression of key genes. Furthermore, fucoidan treatment helps maintain high energy levels by regulating the activities of enzymes related to mitochondrial metabolism, thus effectively improving the resistance of cold-stored cucumber fruits to low-temperature stress and reducing the symptoms of cold injury.

## Data availability statement

The original contributions presented in the study are included in the article/**Supplementary Material**. Further inquiries can be directed to the corresponding author.

## Author contributions

DL: Conceptualization, methodology, software, writing—original draft preparation. RY: validation, formal analysis, data curation, writing—original draft preparation. MX: investigation, resources. SL: visualization, data curation. JC: writing—review and editing, supervision. ZG: writing—review and editing, project administration, funding acquisition. All authors contributed to the article and approved the submitted version.
